# Systematic Analysis of FKBP Inducible Degradation Domain Tagging Strategies for the Human Malaria Parasite *Plasmodium falciparum*

**DOI:** 10.1371/journal.pone.0040981

**Published:** 2012-07-16

**Authors:** Mauro Ferreira de Azevedo, Paul R. Gilson, Heloisa B. Gabriel, Roseli F. Simões, Fiona Angrisano, Jacob Baum, Brendan S. Crabb, Gerhard Wunderlich

**Affiliations:** 1 Departamento de Parasitologia, Instituto de Ciências Biomédicas, Universidade de São Paulo, São Paulo, Brasil; 2 The Macfarlane Burnet Institute for Medical Research and Public Health, Melbourne, Victoria, Australia; 3 Monash University, Melbourne, Victoria, Australia; 4 The Walter and Eliza Hall Institute of Medical Research, Melbourne, Victoria, Australia; 5 University of Melbourne, Victoria, Australia; Weill Cornell Medical College, United States of America

## Abstract

Targeted regulation of protein levels is an important tool to gain insights into the role of proteins essential to cell function and development. In recent years, a method based on mutated forms of the human FKBP12 has been established and used to great effect in various cell types to explore protein function. The mutated FKBP protein, referred to as destabilization domain (DD) tag when fused with a native protein at the N- or C-terminus targets the protein for proteosomal degradation. Regulated expression is achieved via addition of a compound, Shld-1, that stabilizes the protein and prevents degradation. A limited number of studies have used this system to provide powerful insight into protein function in the human malaria parasite *Plasmodium falciparum*. In order to better understand the DD inducible system in *P. falciparum*, we studied the effect of Shld-1 on parasite growth, demonstrating that although development is not impaired, it is delayed, requiring the appropriate controls for phenotype interpretation. We explored the quantified regulation of reporter Green Fluorescent Protein (GFP) and luciferase constructs fused to three DD variants in parasite cells either via transient or stable transfection. The regulation obtained with the original FKBP derived DD domain was compared to two triple mutants DD24 and DD29, which had been described to provide better regulation for C-terminal tagging in other cell types. When cloned to the C-terminal of reporter proteins, DD24 provided the strongest regulation allowing reporter activity to be reduced to lower levels than DD and to restore the activity of stabilised proteins to higher levels than DD29. Importantly, DD24 has not previously been applied to regulate proteins in *P. falciparum*. The possibility of regulating an exported protein was addressed by targeting the Ring-Infected Erythrocyte Surface Antigen (RESA) at its C-terminus. The tagged protein demonstrated an important modulation of its expression.

## Introduction

Malaria is still one of the most important parasitic diseases affecting mankind, with more than 40% of the world’s population at risk of infection [1]. The deadliest form of malaria, caused by *Plasmodium falciparum*, is responsible for almost a million deaths every year, most of them children under 5 years of age (WHO report 2010). Despite global efforts towards control and elimination of the disease, parasite resistance to nearly all drugs has emerged. Realizing the goals of future control efforts will, as such, depend on our ability to quickly diagnose and treat the infected patients and to control disease transmission.

A critical stage in the advancement of novel therapies is developing a means to understand specific aspects of *Plasmodium* biology, such as metabolic or signalling pathways and other fundamental mechanisms of parasite cell or molecular processes. Since *Plasmodium* is not amenable to RNAi based methods [2], the identification of gene function has traditionally relied on classical reverse genetics approaches [3], [4], [5]. Using such approaches, the specific function of a protein is determined by phenotype analysis of null mutant parasites, where the gene encoding the protein of interest is knocked out either via single crossover-mediated homologous integration [6] or by double crossover homologous integration [7]. The haploid nature of the *Plasmodium* asexual blood stage genome has only permitted mutagenesis to be conducted for non-essential *P. falciparum* proteins expressed during this stage [8], [9]. These classic reverse genetic approaches have been particularly useful for studying erythrocyte invasion for the parasites which employ multiple invasion pathways using several ligands none of which by themselves are essential [9]. Genes necessary for life cycle stages other than the asexual blood stage have been successfully studied because the mutants can be made in the blood stages and then passaged through insect and liver stages to obtain phenotypes [10], [11], [12]. However, it is blood stage infection that produces the symptoms of malarial disease and study of this important life cycle stage has been severely hampered by the inability of knockout approaches to functionally dissect the role of essential proteins.

In order to address the function of proteins refractory to deletion, several inducible systems have been developed where the expression of the protein of interest can be modulated and the phenotypes analysed [13]. One of the most promising systems, utilizing conditional regulation of the candidate gene/protein at the post-translational level, was established by generating mutants of the human protein FKBP12 that are unstructured and consequently targeted for degradation in the proteasome. This mutant protein, called a destabilization domain or DD is fused to the protein of interest at its N- or C-terminus, which targets the whole fusion protein for degradation. Regulation is then achieved by adding a cell-permeable ligand, Shld-1 [14], which stabilizes the unstructured protein domain preventing it from protein degradation. The system is functional in several cell types and organisms [15], [16], including *P. falciparum*, where it has been used to test the function of several essential proteins [17], [18], [19]. A related system has also been developed using mutated versions of the *E. coli* DHFR protein, which is unstable when not bound to the drug trimethoprim [20]. Although such a system has been successfully established for *P. falciparum*, it can only be applied to parasites that already express human DHFR since wild type parasites are sensitive to TMP [21].

The attraction of inducible systems for investigating protein function is their ability to specifically inhibit a protein in a regulated and reversible manner. This can be achieved by two basic approaches: 1) conditional knockdown where the protein of interest is expressed at markedly reduced levels; or 2) the regulated expression of a dominant negative variant where modified proteins or parts of proteins are expressed that then inhibit the native target protein or its ligand. In *P. falciparum*, due to the paucity of selectable markers and the long time required to generate stable transfectant parasites, the most straightforward strategy for conditional protein regulation is to integrate a plasmid containing the DD domain into the desired locus via single or double crossover recombination, so that the protein of interest is expressed in fusion with DD at its C-terminus (with the gene remaining under the control of the endogenous promoter). Such an approach, however, may be hindered by considerable leakiness of C-terminal DD tagging that can lead to insufficient target protein degradation and knockdown. In order to improve the strength of knockdown, two new DD variants, referred to as DD24 (E31G-R71G-K105E) and DD29 (D79G-P93S-D100R), have been developed and these provide more efficient and less leaky protein degradation [22].

Conditional knockdown of a *P. falciparum* calpain [19] and a calcium dependent protein kinase [18] have been achieved by C-terminal tagging with either the original DD (L106P) or the new mutant DD29. To our knowledge, these are the only two essential proteins that have been successfully targeted using C-terminal DD tagging with many others failing to accept a C-terminal tag or failing to regulate despite successful integration of either a DD or a DD29 tag (Baum et al. and Azevedo et al., *unpublished data*). The inability of different proteins to accept a C-terminal tag or the failure of the stabilizing ligand to restore expression levels that allow parasites to grow normally likely accounts for the lack of success to achieve integrated regulation. Variation in the ability to successfully regulate those proteins that will accept a tag likely arises because the degradation seen is not sufficient or complete enough to cause a detectable phenotype (incomplete degradation). Conditional expression of proteins that produce dominant-negative phenotypes has not yet been reported for *P. falciparum*.

To gain a better understanding of the performance of the available DD tagging strategies, we have conducted a systematic approach to measure the toxicity of Shld-1 as well as the activities of the available DD variants in *P. falciparum*. The single original mutant L106P, referred to as DD, has been characterized [17], [19], as well as the triple mutant DD29 [18]. The triple mutant DD24 has only been characterized in other cell types [22]. Here, using the reporter proteins luciferase (Luc) and GFP, we show that when tagged to the C-terminal, DD24 performs clearly better than the other mutants regarding protein regulation at Shld-1 concentrations that are tolerated by the parasite. In contrast, N-terminal tagging with either of the triple mutants was less efficient compared to the original DD single mutant. The possibility of using the system with secreted or exported proteins was addressed by C-terminally tagging the endogenous gene for the exported *Ring Infected Erythrocyte Surface Antigen* (RESA) with DD24. Successful regulated expression and export of RESA-DD demonstrate that proteins secreted via the endoplasmic reticulum (ER) can be regulated.

## Results

### Effect of the Shld-1 on Parasite Growth

The application of the DD system includes the incubation of parasite forms with the small stabilizing molecule Shld-1 which is considered non-toxic to *P. falciparum* at the concentrations used in other cell systems. However, it is possible that Shld-1 interferes subtly with *Plasmodium* cell cycle progression or proliferation leading to a misinterpretation of eventual phenotypes. *P. falciparum* conditional knockdown or knockout lines have been successfully generated [18], [19] when parasites had been maintained in 0.5 µM Shld-1. Depending on the target protein tagged, it may be desirable or necessary to increase the concentration of Shld-1. In order to quantify the toxicity of Shld-1 to the parasites and to determine whether a higher concentration would be tolerable, *P. falciparum* ring stage parasites were incubated with either 0.5 µM or 1.0 µM of Shld-1 for up to four days. Parasite growth was measured by flow cytometry and parasite forms were analysed by microscopy of blood smears.

**Figure 1:**
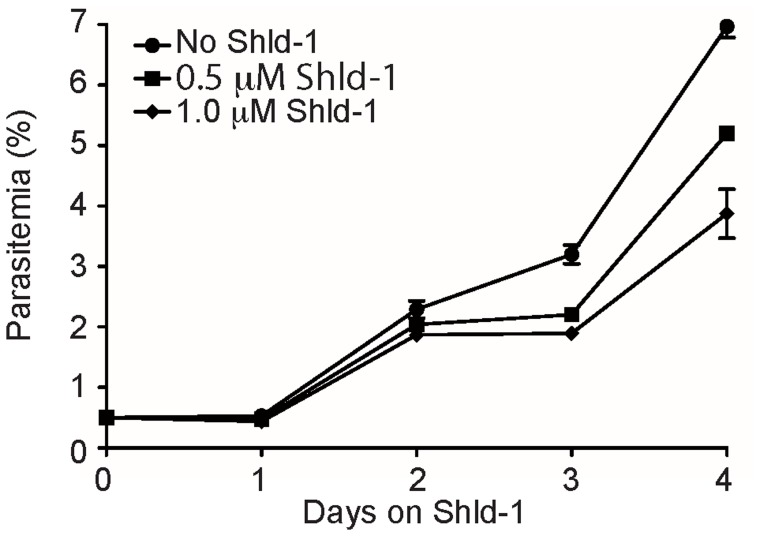
Effect of Shld-1 on parasite development. *P. falciparum* 3D7 blood stage parasites were incubated with or without the indicated concentrations of Shld-1 and the parasitemia monitored for 4 days by flow cytometry.

Parasitemias of the cultures kept at 0.5 µM and 1.0 µM Shld-1 were reduced by 11% and 18% after the first reinvasion cycle (48 hours) and by 25% and 45% after the second cycle, respectively, compared to no Shld-1 controls (Fig. 1). A subtle delay in the trophozoite development could be made out over one blood stage cycle when treating with Shld-1, while the incubation for 24 h did not reveal differences between treated or untreated parasites (Fig. S3). To ascertain if *P. falciparum* could tolerate being cultured in 1.0 µM Shld-1 for a longer period, parasites were further grown for several weeks and continued to expand, proving that despite its apparent toxicity, 1.0 µM could be employed for long term growth (data not shown).

### Vectors for Inducible Expression of Proteins in *P. falciparum*

Prior studies and our own work have demonstrated that N-terminal tagging of proteins with a Destabilization Domain (DD) tag provides a much greater efficiency of destabilization than C-terminal tagging [14], [17] (Fig. S1). This observation has proven valid for a variety of cell types, including *P. falciparum*, which may explain why reversible phenotypes of only two conditional knockout lines have been reported [18], [19].

**Figure 2:**
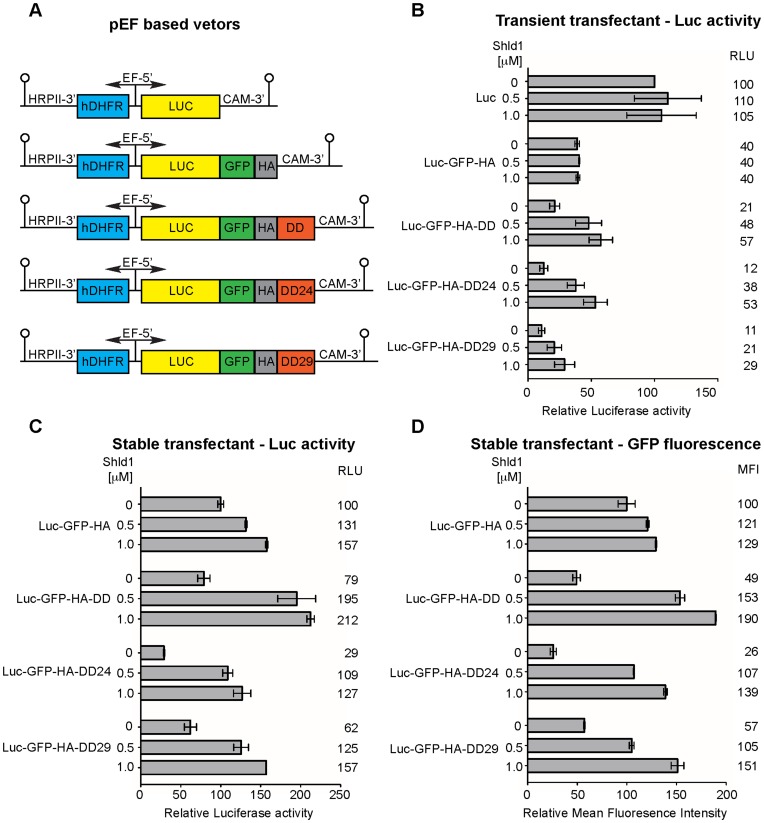
Regulation with C-terminal DD. (A) Maps of inducible expression vectors. (B) To measure expression in transiently transfected parasites ring stage parasites were transfected and the indicated Shld-1 concentrations were added the next day and maintained for 24 hours. Luciferase expression is represented relative to the positive control pEF-Luc kept without Shld-1. Results indicate the average of 4 experiments and error bars show the standard deviation. (C) Luciferase activity on stably transfected parasites. Ring stage parasites of stable transfected lines were incubated for one day with the indicated Shld-1 concentrations. The measured luciferase activity was normalized by the number of parasites and expressed as percentages to the control line 3D7 transfected with Luc-GFP kept without Shld-1. (D) GFP fluorescence of the parasites described in (C) was measured by flow cytometry and is expressed relative to the control line Luc-GFP kept without Shld-1. RLU – relative light units, FI – fold induction of reporter expression, MFI – mean fluorescence intensity.

To explore this concept further, a rapid read out for the effects of DD and Shld-1 on the expression of a reporter gene encoding *Photinus* luciferase (Luc) was undertaken with *P. falciparum* blood stage parasites. Plasmids encoding *luc* fusions were electroporated and luciferase activity was measured one day later. GFP was also incorporated into some of our Luc fusion proteins so that an option to check the expression of the fusion proteins by fluorescence microscopy or flow cytometry was available. A plasmid encoding luciferase alone and luciferase-GFP tagged with a triple haemagglutinin epitope (HA) served as positive controls for transfection efficiency and enzyme activity (pEF-Luc & pEF-Luc-GFP-HA, Fig. 2A). In addition to the originally described and functional single DD mutant (L106P) (Fig. S1), we made a Luc-GFP-HA gene fusion with the DD24 and DD29 triple mutants. The three DD containing plasmids were accordingly called pEF-Luc-GFP-HA-DD/DD24/29 (Fig. 2A).

### Regulation in Transiently Transfected Parasites

The luciferase reporter plasmids were transiently transfected in *P. falciparum* and the parasites were cultured in the presence of 0, 0.5 or 1.0 µM Shld-1 for one day after which they were harvested and their luciferase activity determined (Fig. 2B). Importantly, in the sample without Shld-1, the culture medium was adjusted to 0,1% ethanol. Also, the incubation time with Shld-1 was limited to 24 h in which no toxic inhibitory effect on parasite progression is observed (Fig. 1 and Fig. S3). In the absence of Shld-1 the presence of the GFP-HA reduced luciferase activity to 40% of the luciferase only control. The DD further reduced reporter activity to about only 21% of pEF-Luc (Fig. 2B). The reporter activity of parasites transfected with the DD24 and DD29 plasmids was only 12% and 11% respectively, and much lower than the activity detected with the original DD plasmid. This indicates that in *P.* *falciparum* the DD triple mutants destabilize the proteins approximately twice as efficiently as the original DD.

In the presence of 0.5 and 1.0 µM Shld-1 the luciferase activity of pEF-Luc and pEF-Luc-GFP transfected parasites was almost the same as without the compound (Fig. 2B), excluding any nonspecific effect of Shld-1 upon luciferase activity which can be observed after longer Shld-1 incubations (Fig. 1). In contrast, cultures transfected with each of the DD vectors and kept in 0.5 µM or 1.0 µM Shld-1 always showed increased luciferase activity when compared to the same transfected parasites kept in the absence of the ligand. In 0.5 µM and 1.0 µM Shld-1, the reporter activity relative to the control was increased to a respective 48% and 57% for the DD, 38% and 53% for DD24 and to 21% and 29% for DD29 (Fig. 2B). The culturing of DD and DD24 transfectants on 1 µM Shld-1 elevated the luciferase activities to a level exceeding the pEF-Luc-GFP-HA control parasites. In contrast, the luciferase activity of parasites transfected with DD29 plasmid was only raised to half of the Luc-GFP-HA parasites in 1 µM Shld-1.

### Regulation in Stably Transfected Parasites

To test whether the performance of the DD mutants would be maintained in transfectants containing the luciferase and GFP genes in episomes, parasites were stably transfected with the DD reporter plasmids. Reporter expression was then induced similarly to the transient transfection experiments, except that Shld-1 was added to ring stage parasites. Luciferase reporter activity of non-induced parasites suggested DD24 is the most efficient domain in destabilizing luciferase, followed by DD29 and DD (Fig. 2C). This result is somewhat different to the transient transfection data where both DD24 and DD29 parasites destabilised luciferase to the same degree (Fig. 2B). The other difference was that in the transient data Shld-1 drug restored greater luciferase activity in DD24 compared to DD29 mutants but in the stably transfected parasites restoration was similar (Figs. 2A & B). Although luciferase activity was normalized by parasite number in the stable transfectants, small differences in parasite cell cycle synchronization could have affected reporter activity. To further ensure synchronisation was not contributing to the differences, GFP fluorescence of the stable transfectants was quantified by flow cytometry, where cells were gated on late stage parasites. DD24 still destabilized luciferase to the lowest levels, followed now by DD and DD29. The effect of Shld-1 was similar to what was detected using the luciferase assay (Fig. 2D). The results calculated as fold induction upon Shld-1 treatment are summarized in Table 1.

**Table 1:** Reporter proteins - regulation summary.

	Shld-1 induction (fold change)							
	Transient - Luc		Stable - Luc		Stable - GFP		Average	
Reporter	0.5 µM	1.0 µM	0.5 µM	1.0 µM	0.5 µM	1.0 µM	0.5 µM	1.0 µM
**C-DD**	2.28	2.71	2.46	2.68	3.12	3.87	2.62	3.08
**C-DD24**	3.16	4.41	3.75	4.37	4.11	5.34	3.67	4.70
**C-DD29**	1.90	2.63	2.01	2.53	1.84	2.64	1.91	2.60
**N-DD**	2.90	3.00	ND	ND	ND	ND	ND	ND
**N-DD24**	1.63	1.45	ND	ND	ND	ND	ND	ND
**N-DD29**	1.66	1.50	ND	ND	ND	ND	ND	ND

C- refers to Luc-GFP-HA fused to the indicated DD mutant at its C-terminus and N- to Luc fused to DD at its N-terminus. Fold change represents the means of the expression of the Shld-1 induced related to the basal (non induced) expression. See figure 2 for relative expression values.

Despite some differences between luciferase/GFP expression in the transient versus stable transfectants, which may also have been due to plasmid copy number in the latter, the DD24 tagged line consistently showed the highest fold induction (difference between induced and non-induced state) in each test when either 0.5 µM (3.2–4.1 x) or 1.0 µM (4.4–5.3 x) Shld-1 was used. DD had the second best induction followed by DD29 (Fig. 2B-D, Table 1). Notably, the plasmid copy numbers as evaluated by quantitative PCR reactions using equally performing primers for a single copy gene (seryl-t-RNA synthetase, PlasmoDB PF07.0073) and luciferase showed a comparable number of plasmid equivalents in the transfectants (Fig. S4). Assuming that the copy number of plasmids is not decreasing dramatically during Shld-1 removal/addition, our results indicate that the observed differences between the different DD mutants occurred as a consequence of the differing DD domain sequences and not due to transcriptional differences caused by vastly differing plasmid copy numbers in transfectants.

### Performance of the DD Mutants for N-terminal Tagging

**Figure 3:**
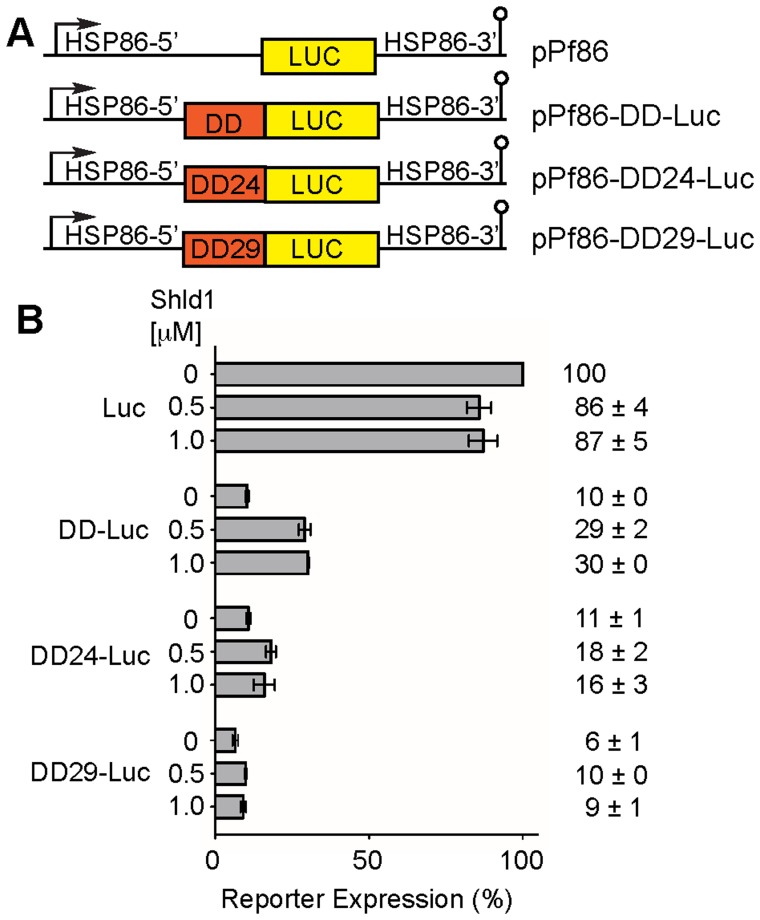
Regulation with N-terminal DD. (A) Maps of reporter plasmids tested. (B) Reporter expression of transiently transfected parasites. Parasite transfection and Shld-1 incubation was performed as indicated in Figure 2. Luciferase activity is represented relative to pPf86 kept without Shld-1. Results are the average of 3 experiments and the error bars show the standard deviation.

Considering the reproducibly improved regulation of protein levels achieved with DD24 when expressed at the C-terminal of the reporter proteins, the regulation of N-terminal tagging was also investigated. Since the reporter activity obtained in *P. falciparum* transient transfections is somewhat low and DD degrades proteins more efficiently when fused to its N-terminus (Fig. S1), reporter plasmids were constructed based on the pPf86 vector [23], where luciferase is under the control of the HSP86 promoter, which is about ten times stronger than the EF1-α promoter (Fig. 3A).

The N-terminal DD reduced luciferase activity 10 fold, confirming the more efficient protein destabilization (Fig. 3B). Shld-1 partially prevented the degradation restoring the activity to 29% and 30% when cultures were kept on 0.5 µM and 1.0 µM Shld-1, respectively (3 fold induction). The triple mutant DD24 destabilized luciferase as efficiently as DD, but reporter activity was poorly reverted by Shld-1, 18% and 16%, respectively (∼1.5–1.6 fold induction). DD29 was an even more efficient destabiliser, reducing reporter activity even further to about 6%, but similarly to DD24, reporter expression poorly recovered when Shld-1 was added, increasing to 10% and 9%, respectively.

### DD System can Regulate the Expression of Exported Proteins

**Figure 4:**
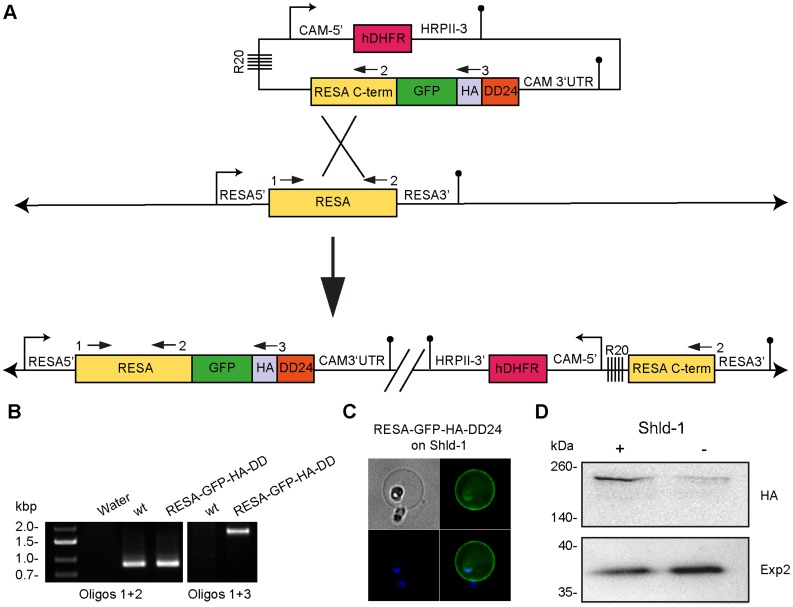
DD can be used to regulate exported proteins. (A) Diagram of DNA integrations into the RESA locus to produce the transgenic line. After integration in the presence of Shld-1, RESA was expressed in fusion with the GFP-HA-DD24 under the control of the native RESA promoter. (B) Integration of the plasmid in the RESA endogenous locus is confirmed by PCR. The oligonucleotides used are indicated by arrows in (A). (C) Fluorescent microscopy of live parasites labelled with DAPI showing RESA-GFP-HA-DD24 is exported to the RBC membrane. (D) Western blot analysis of RESA-GFP-HA-DD24 transfectants showing that RESA levels decrease after the removal of Shld-1. Parasites integrated in the presence of Shld-1 at ring stage were split and kept either with 0.5 µM or no Shld-1 for 2 days when RBC ghosts were collected and protein were fractionated by SDS-PAGE. The western blot was probed with anti-HA mAb to detect RESA-GFP-HA-DD24. As the loading control, the western blot probed with polyclonal rabbit anti-Exp2 [31].

While proteins localized in the parasite cytoplasm have been successfully regulated by Shld-1, it was uncertain whether proteins that are secreted via the ER or exported to the red blood cell could still be destabilized. In order to investigate the efficacy of the system to regulate an exported protein, RESA was tagged with GFP-HA-DD24 at its C-terminus (Fig. 4A). Parasites were transfected and then kept in 0.5 µM Shld-1 and the transfected cultures were intermittently subjected to WR99210 withdrawal to select for plasmid integration. The resulting parasites were then analysed by PCR using oligonucleotides that could only amplify a product if integration had occurred (Fig. 4B). Images of fluorescence live microscopy confirmed that RESA was expressed in fusion with the tag at its expected localization, associated to the host cell membrane (Fig. 4C, Fig. S2).

We then monitored the regulation removing the Shld-1 from ring stage cultures for 2 days after which ghosts of infected RBC were fractionated and proteins extracted with SDS. Western blots probed with anti-HA antibody showed greatly reduced levels of RESA-GFP-HA-DD24 in parasites where Shld-1 had been removed (Fig. 4D). Importantly, loading controls with EXP2 (a component of the putative parasite export machinery co-purifying with IRBC membranes [24]) demonstrated the reduction of RESA-GFP-HA-DD24 was due to its DD-mediated degradation and not due to whether cultures were subjected to Shld-1 (Fig. 4D). Densitometry analysis of the bands detected in the Western blot suggests that removal of Shld-1 resulted in about 4 fold less tagged RESA protein (data not shown). Fluorescent live microscopy imaging also showed reduction in RESA-GFP-HA-DD24 expression in parasites where Shld-1 had been removed. Moreover, GFP fluorescence was more intense inside the parasite, suggesting a partial degradation prior to insertion in the endoplasmic reticulum although the resolution does not permit more specific affirmations (Fig. S2).

## Discussion

The two main approaches applying inducible systems to understand gene function are i) the conditional knockdown and ii) the expression of proteins that produce a dominant negative phenotype. In the former, the proteins of interest must be stabilized during selection, such as with tolerant Shld-1 concentrations, for periods up to many months. In the second strategy, the expression of proteins or peptides, supposed to be toxic or deleterious, must be kept silent, so Shld-1 has to be added to parasites already selected for the presence of the plasmids in the form of episomes. The conditional knockouts of a protease [19] and a kinase [18] have been conducted this way, selecting parasites on 0.5 µM Shld-1. This enabled the generation of cloned cell lines where the target proteins were regulated at sufficient levels to produce inducible and reversible deleterious phenotypes providing important clues of their respective functions. According to our results, the concentration of 0.5 µM Shld-1 is slightly toxic to the parasites, reducing their growth to about 11% per reinvasion cycle. We also showed that although parasites tolerate up to 1 µM of Shld-1, their development is delayed and this must be taken in account when interpreting inducible phenotypes. We could not assign the Shld-1 growth defect phenotype to the inhibition of a specific intraerythrocytic stage or to RBC invasion (Fig. S3). Therefore, it remains to be shown whether unique or multiple parasite proteins are natural targets for Shld-1. A *Plasmodium* FKBP homologue is expressed throughout the life cycle and it is sensitive to the Shld-1 analogue Rapamycin [25], but whether Shld-1 targets PfFKBP is unknown.

In this study we present an in depth-analysis of the most useful system currently available to achieve controlled protein expression in *Plasmodium*. While regulation of protein levels by the DD system in *P. falciparum* has been demonstrated using a small number of proteins, we show here the potential of the technique using transiently, stable and integrated plasmid constructs containing three different variants of the original FKBP (DD) domain. Similar to what has been characterized for NIH3T3 cells [22], data from transiently and stably transfected parasites, using either Luc or GFP as reporters, suggested that DD24, and possibly DD29, are more efficient than the original DD in the destabilization of proteins when fused to their C-terminus. Also, at Shld-1 concentrations which are tolerated by *P. falciparum* cultures, DD24 has the greatest dynamic range of the three DDs tested. While our data generally agree with what has been described for the DD mutants in other cell types, the destabilization efficiency and the dynamic range of induction observed in *P. falciparum* are quite low compared to what Chu et al. [22] measured. It was shown that in NIH3T3 cells the DD24 or DD29-tagged reporter proteins were reduced to about 5% of the control lines; however, in *P. falciparum*, the reduction was to about 10–20% depending on the control used. In the same way, induction with 1 µM Shld-1 increased protein levels about 20 fold against 4–5 fold in *P. falciparum*. Importantly, our values were obtained in quantifications using two different reporter proteins, and both showed similar induction performance. This suggests that the regulation provided by the DD system, despite evidence for a considerable regulation, is generally lower in *P. falciparum*. Longer Shld-1 incubations were tried, but resulted in only slightly better induction and no more than 6 fold in 4 days was attained. Higher Shld-1 concentrations were tested, but they were too toxic to the parasite. It is not clear whether these differences are due to less active protein degradation in parasites compared to NIH3T3 cells.

Although DD24 seems to be the mutant with the best regulation, DD29 has been successfully applied in the CDPK5 conditional knockout [18], indicating the regulation obtained with this mutant can be sufficient to cause an inducible lethal phenotype. It is possible though, that DD24 could be successfully used to regulate proteins that cannot be targeted with DD29 because they need to be expressed at higher levels to guarantee the survival of parasites.

Tagging the proteins with DD at the N-terminus had been reported to produce more efficient protein destabilization both for the apicomplexan parasites *P. falciparum* [17] and *Toxoplasma gondii* [15] and also for other eukaryotic cell lines [14]. We investigated whether the triple mutants would allow the protein to be further degraded. While the reporter activity was equally (DD24) or more efficiently (DD29) reduced than with the classic DD, expression was poorly restored with Shld-1, suggesting the triple mutants are not suitable for N-terminal tagging, probably by rendering the reporter protein unstable.

*Plasmodium* parasites have unique cellular compartments where DD tagged proteins might not be as efficiently targeted for degradation as those that reside in cytosolic environments. For example, it is unclear if proteins directed to the nucleus or to the apicoplast can be efficiently DD-tagged and degraded which may depend on specific functional features of the target proteins. It is possible that proteins become non-functional despite of being normally transported to their target organelle in the presence of a stabilized DD domain. On the other hand, C-terminally tagged proteins with co-translational insertion in the endoplasmic reticulum may escape from proteasome degradation in the absence of Shld-1. Since a number of potential virulence factors are exported from the parasite, we addressed the feasibility of regulating RESA, a protein that is exported to the host cell membrane during erythrocytic stages. Removal of Shld-1 from parasites caused a clearly visible reduction in the levels of RESA present in the RBC compartment. Since the period from which Shld-1 was removed until protein samples were acquired was about one reinvasion, it seems that proteins synthesized in the absence of Shld-1 are sensitive to degradation and it is probable that the punctuate weak fluorescent signal inside the parasite was caused by GFP *en route* to degradation. It still remains to be determined what happens to tagged RESA that has already been exported and if it is sensitive to degradation. Recently, the 20S proteasome subunit was identified in human mature erythrocytes [26], turning possible that even exported destabilized proteins may be degraded by the erythrocyte proteasome.

### Conclusion

In conclusion, our study demonstrates the possibilities and limits in which proteins can be modulated using the FKBP-mutants DD, DD24 and DD29 and establishes the groundwork for experimentation using this system on virtually any target in the *Plasmodium* proteome. This includes the many proteins resident in organelles or which function in pathways that lead to the secretion of proteins to the host cell and which may prove novel targets for intervention against malaria disease.

## Materials and Methods

### Plasmid Construction

The plasmid pTGFP [27] was digested with *Xho* I and re-ligated to delete the transactivator and its 3′UTR. GFP was PCR amplified from the same plasmid with the oligonucleotides 5′-ctcgagctgcagaaaaaatggctacacgtgca and 5′-actagtacgcgttgctttgtatagttcat and cloned back in the vector digested with *Xho* I and *Spe* I, generating the plasmid pRM2-GFP. This plasmid incorporates the rep20 element for efficient segregation during mitosis [28] and has the selection and expression cassettes cloned back to back, where GFP is under the control of the MSP2 promoter. Plasmid templates for DD variants were kindly provided by Thomas Wandless (Stanford University, USA). DD combined with a PCR amplified triple haemagglutinin (HA) tag were fused by PCR using the oligonucleotides 5′-acgcgtccgtacgacgtc and 5′-actagtttattccggttttagaagc and cloned in pRM2-GFP digested with *Mlu* I and *Spe* I, generating pRM2-GFP-DD. DD24 and 29 were amplified from plasmids pYFP-E31G-R71G-K105E (24) and pYFP-D79G-P93S-D100R (29), using the forward oligonucleotide 5′-gctagcatgggagtgcaggtggaaac and the reverse oligonucleotides 5′-actagttattccagttctagaagctccac (24) or 5′-actagttattccagttttagaagctccac (29) and cloned in pRM2-GFP-DD, generating pRM2-GFP-DD24 and pRM2-GFP-DD29. Both promoters and the Rep20 sequences of these plasmids were replaced with the bidirectional *P. berghei* EF1α promoter, which was PCR amplified from *P. berghei* gDNA using the oligonucleotides 5′-gctctagaggatccttttataaaatttttatttatttataagca and 5′-ctcgagttttataaaatttttatttatttataagca and cloned in pBluescript (Stratagene). The restriction sites *Spe* I and *Hind* III were destroyed by digesting the plasmid with these enzymes and then filling the ends with Klenow and re-ligating them. The promoter was then digested with *Bam* HI and *Hind* III and cloned in pRM2-GFP-DD/24/29, generating pEF-GFP-DD/24/29. Luciferase was amplified from pGL3 (Promega) with oligonucleotides 5′-ctcgaggtcccatggaagacgccaaaaaca and 5′-actagttgctgcagccacggcgatctttccgc and cloned in pEF-GFP-DD/24/29 digested with *Xho* I and *Spe* I or *Pst* I, generating pEF-Luc and pEF-Luc-GFP-DD/24/29 respectively. The GFP-HA-DD sequence of pEF-Luc-GFP-DD was replaced with GFP-HA digesting the vector with *Pst* I and *Spe* I and the insert retrieved from pRM2-GFP-DD digested with *Pst* I and *Nhe* I, generating pEF-Luc-GFP-HA.

The DD-Luc was fused by PCR with oligonucleotides 5′-ccatgggagtgcaggtggaa and 5′-gcgtcttcctgcagttccggttttagaagctcca (DD) and 5′-accggaactgcaggaagacgccaaaaacataaaga and 5′-accggaactgcaggaagacgccaaaaacataaaga (Luc), digested with *Nco*I and *Spe*I and cloned in pPf86 digested with *Nco*I and *Xba*I to make pPf86-DD-Luc. DD24 and DD29 were amplified by PCR with oligonucleotides 5′-ccatgggagtgcaggtggaaacca and 5′-ctgcagttccagttctagaagctccaca (DD24) or 5′-ctgcagttccagttttagaagctccacac (DD29), digested with *Nco*I and *Pst*I and cloned in pPf86-DD-Luc to make pPf86-DD24/29-Luc.

### *P. falciparum* Culture and Transfection

Parasites were transfected as previously described [3], using the electroporation conditions established elsewhere [29]. Briefly, *P. falciparum* 3D7 was cultured in 4% hematocrit in RPMI-HEPES supplemented with 0.5% Albumax 1 (Invitrogen). Ring state parasites at 5–8% parasitemia were transfected with 75 µg (transient) or 150 µg (stable) of plasmid. The culture media were changed on the second day and parasites were harvested for reporter assays (transient) or submitted to drug pressure with 2.5 nM WR99210 (stable) on the third day. Stably transfected parasites were cultured in standard conditions until parasites re-appeared and normal growth was re-established.

### Shld-1 Incubation

Shld-1 was diluted in ethanol to a stock concentration of 1 mM and stored in –20°C. Immediately prior to use, it was diluted in RPMI to the indicated concentration. For the transient transfections, parasites were split and Shld-1 added one day after the electroporation. Unless specified, parasites were kept under Shld-1 for one day.

### Luciferase Assays

Parasites were saponin lysed and the pellet washed twice in PBS. After resuspension in 1 x lysis buffer (Promega), the lysed cells were mixed with luciferase assay reagent (Promega) and luciferase activity was measured in the Lumat LB 9507 luminometer (EG & G Berthold). For each experiment, the reporter activity was expressed as the percentage of the activity measured in the positive control, usually parasites transfected with similar luciferase plasmids without DD. The results are the average of at least three independent experiments for transient transfections and of a representative experiment with stable transfected parasites, done in duplicates.

### Detection of Parasitemia by Flow Cytometry

Parasites were labeled with 10 µg/ml ethidium bromide as described previously [30], washed once in PBS and analysed on a Guava cytometer (General Electric).

## Supporting Information

Figure S1 **Efficient Shld-1 regulation of N terminally tagged DD (original) tagged proteins in** ***P. falciparum.*** (A) Plasmid derived from pRM2-GFP for N terminal DD tagging of a reporter gene - here a triple HA tag fused to GFP. This plasmid contains the strong and schizont-specific msp2 promoter. B) Western blot of synchronous schizont lysates transformed with pRM2-DD-3HA-GFP with anti-GFP (1∶1000, Roche) demonstrates efficient Shld-1 dependent regulation by addition of either 0.5 µM or 0.1 µM to parasite ring stages. An asterisk marks a GFP breakdown product. Note that we used a smaller Shld-1 concentration in order to avoid the delay in intraerythrocytic development observed with higher Shld-1 concentrations which would lead to an overall decreased signal due to weaker promoter activity in earlier than schizont stages.
(TIF)

Figure S2 **Fluorescent microscopy of RESA-GFP-HA-DD24 parasites cultured in the presence of Shld-1 (left) or when the ligand had been removed for 2 days (right).**
(TIF)

Figure S3 ***Plasmodium falciparum*** **D10 parasites were highly synchronized by sorbitol and heparin treatment.** Shld-1 (1 µM) was added to either early ring stage (t = 0 h) or trophozoite stage parasites (t = 24 h). Parasite development was monitored by Giemsa-stained blood smears taken every 8 hours for 48 hours.
(JPG)

Figure S4 **Parasites from transfectants had their genomic DNA extracted by standard methods** [**32**] **and the following oligonucleotides were employed to amplify either plasmodial seryl-t-RNA synthetase (PF07_0073, 5′-AAGTAGCAGGTCATCGTGGTT, 5′-TTCGGCACATTCTTCCATAA) or** ***Photinus*** **luciferase (5′-CGTCGCCAGTCAAGTAACAA, 5′-TTTCTTGCGTCGAGTTTTCC) by standard qPCR using Fermentas SYBR realtime PCR mix in an Eppendorf realplex^2^ thermocycler.** Equal primer performance was tested beforehand using plasmids with cloned target sequences. The differences in copy numbers were expressed as 2^−ΔCt^ values (y-axis) which indicate how many times more luciferase target molecules are in the sample in relation to the genomic control seryl-t-RNA synthetase [33]. The four indicated gDNA samples were analysed in three independent experiments using triplicates for each gDNA sample and primer pair and the standard deviation between the experiments is shown.
(TIF)
