# Supplementary figures and images for: Systematic Analysis of FKBP Inducible Degradation Domain Tagging Strategies for the Human Malaria Parasite Plasmodium falciparum
Source: PLoS One. 2012 Jul 16;7(7):e40981. doi: 10.1371/journal.pone.0040981 (PMC3397994; doi:10.1371/journal.pone.0040981)

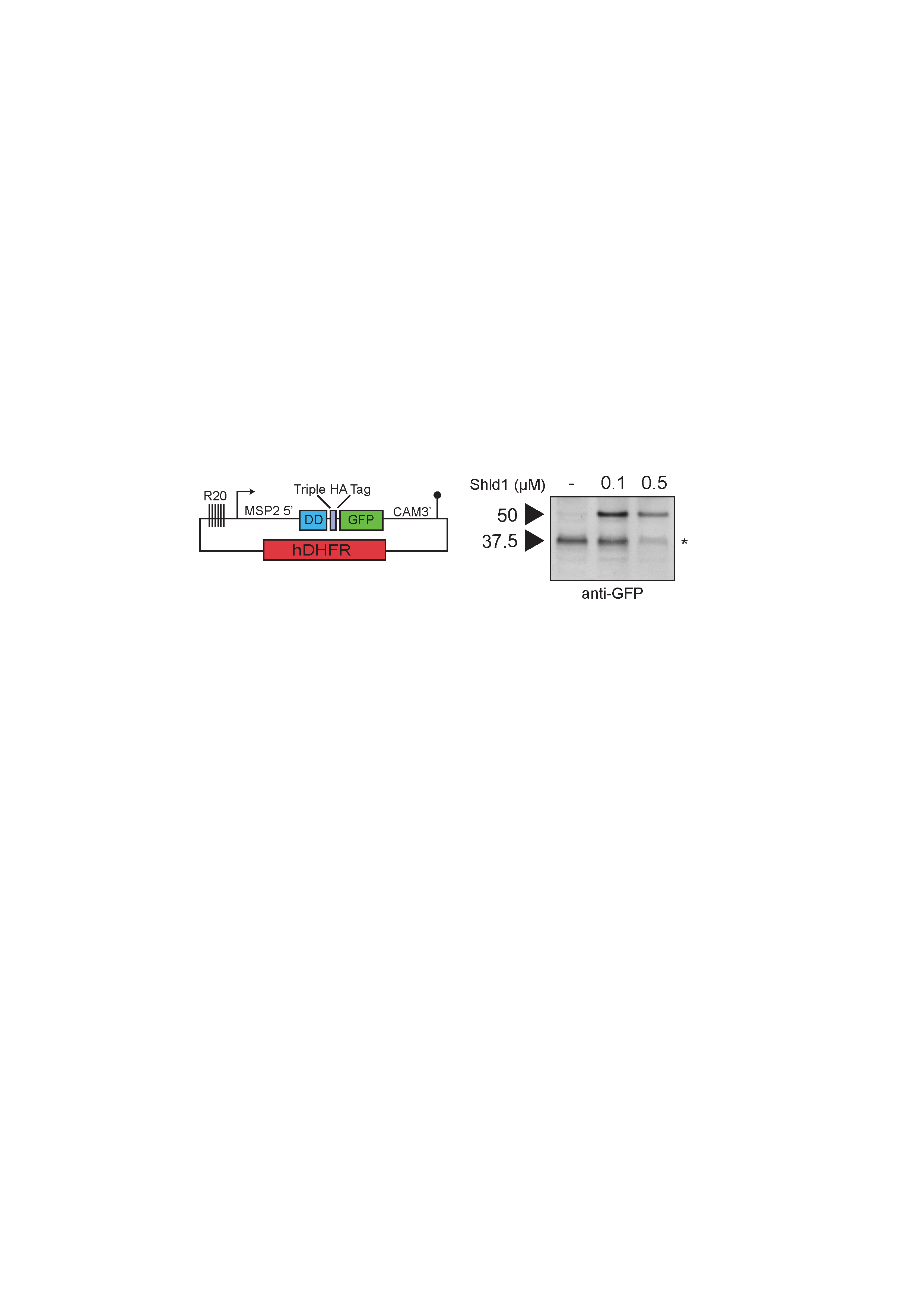

Supplement: Figure S1 — Efficient Shld-1 regulation of N terminally tagged DD (original) tagged proteins in P. falciparum. (A) Plasmid derived from pRM2-GFP for N terminal DD tagging of a reporter gene - here a triple HA tag fused to GFP. This plasmid contains the strong and schizont-specific msp2 promoter. B) Western blot of synchronous schizont lysates transformed with pRM2-DD-3HA-GFP with anti-GFP (1∶1000, Roche) demonstrates efficient Shld-1 dependent regulation by addition of either 0.5 µM or 0.1 µM to parasite ring stages. An asterisk marks a GFP breakdown product. Note that we used a smaller Shld-1 concentration in order to avoid the delay in intraerythrocytic development observed with higher Shld-1 concentrations which would lead to an overall decreased signal due to weaker promoter activity in earlier than schizont stages. (TIF) [file pone.0040981.s001.tif]

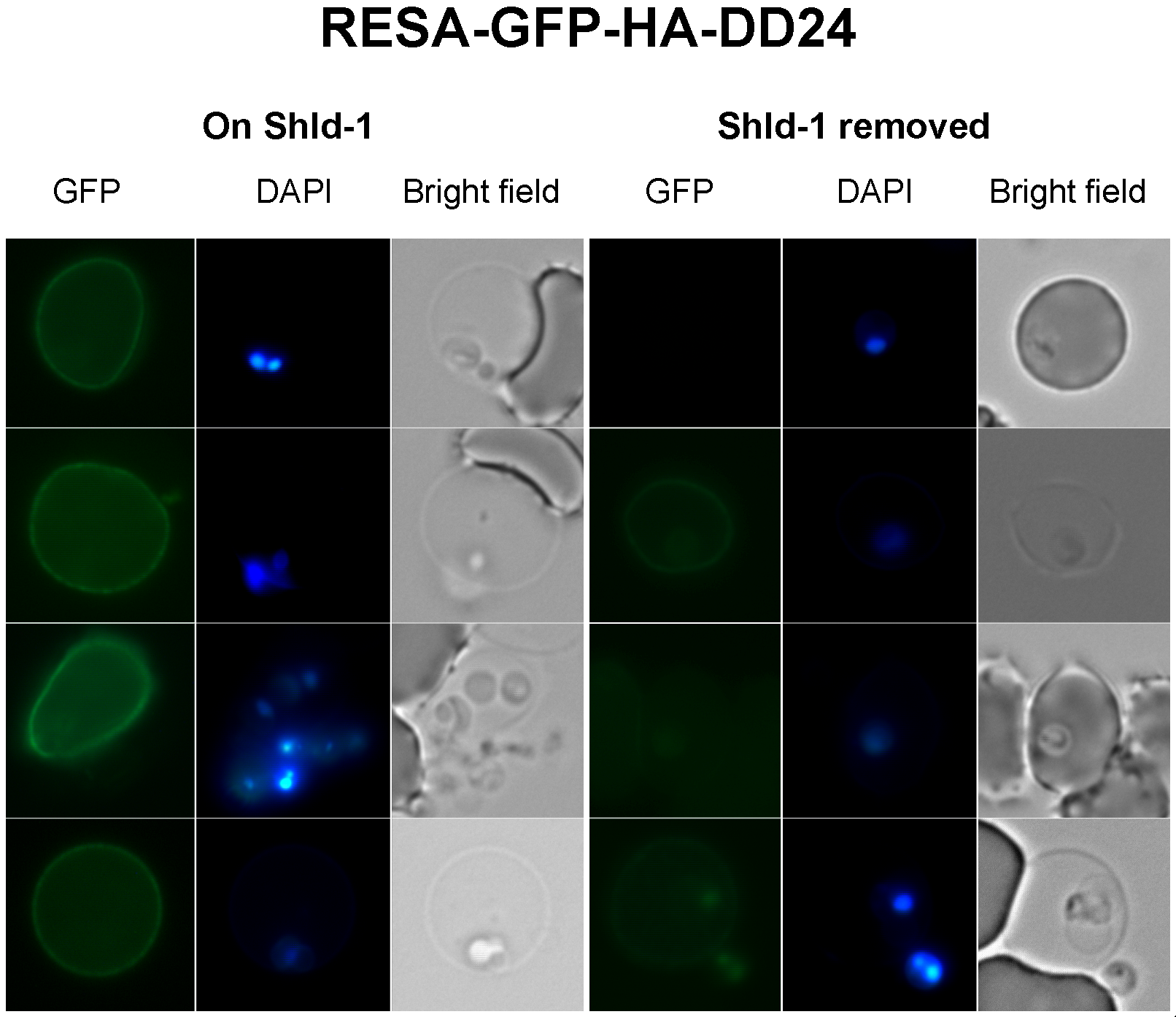

Supplement: Figure S2 — Fluorescent microscopy of RESA-GFP-HA-DD24 parasites cultured in the presence of Shld-1 (left) or when the ligand had been removed for 2 days (right). (TIF) [file pone.0040981.s002.tif]

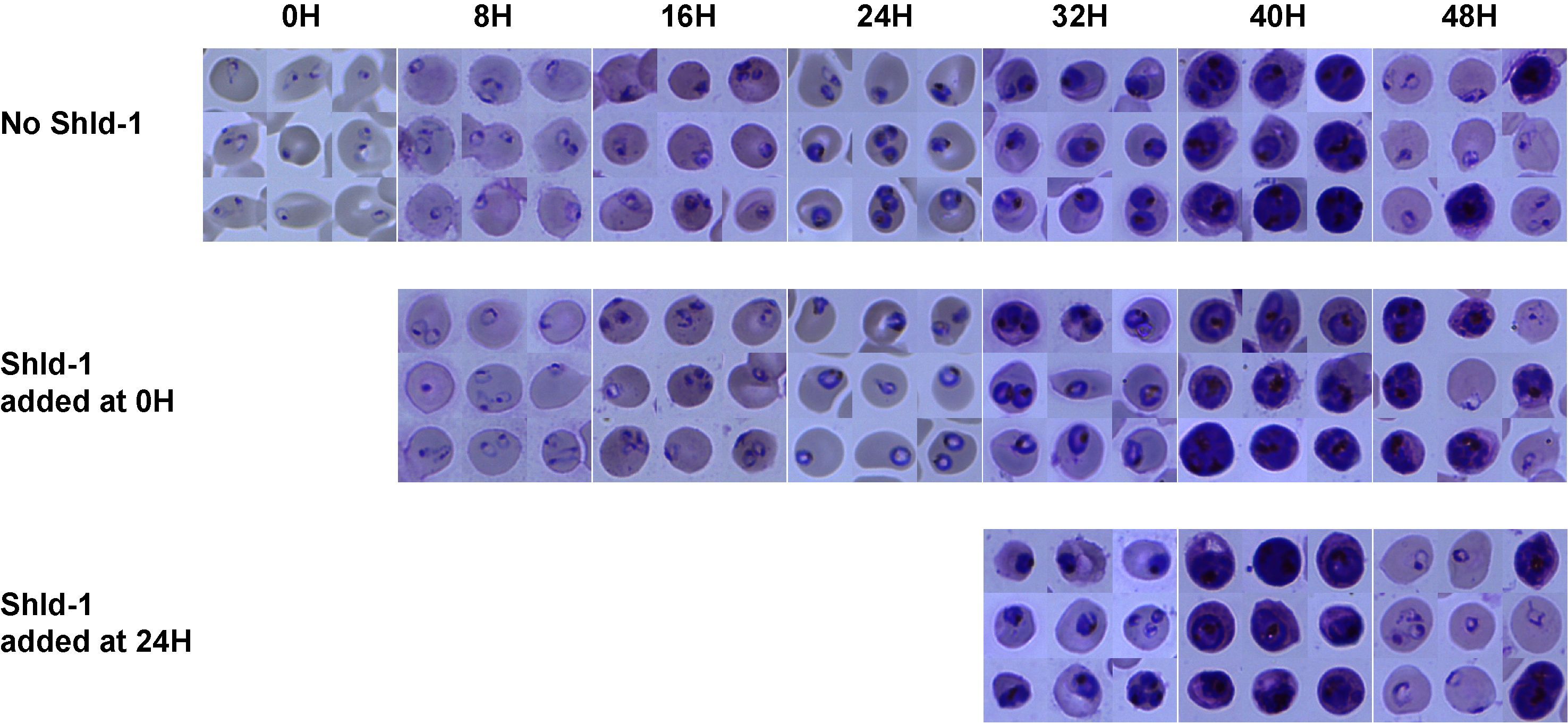

Supplement: Figure S3 — Plasmodium falciparum D10 parasites were highly synchronized by sorbitol and heparin treatment. Shld-1 (1 µM) was added to either early ring stage (t = 0 h) or trophozoite stage parasites (t = 24 h). Parasite development was monitored by Giemsa-stained blood smears taken every 8 hours for 48 hours. (JPG) [file pone.0040981.s003.jpg]
